# Prevalence of ventricular parasystole in patients with cardiac sarcoidosis: correlation between parasystole and inflammation in ventricular fibrillation

**DOI:** 10.1136/openhrt-2025-003196

**Published:** 2025-04-09

**Authors:** Takashi Ikee, Kenzaburo Nakajima, Kohei Ishibashi, Emi Tateishi, Reina Tonegawa-Kuji, Toshihiro Nakamura, Satoshi Oka, Yuichiro Miyazaki, Akinori Wakamiya, Nobuhiko Ueda, Tsukasa Kamakura, Mitsuru Wada, Yuko Inoue, Koji Miyamoto, Satoshi Nagase, Takeshi Aiba, Tetsuya Fukuda, Kengo Kusano

**Affiliations:** 1 Department of Cardiovascular Medicine, National Cerebral and Cardiovascular Center Hospital, Suita, Japan; 2 Department of Radiology, National Cerebral and Cardiovascular Center Hospital, Suita, Japan; 3 Genomic Medicine Institute, Lerner research institute, Cleveland Clinic, Cleveland, Ohio, USA; 4 Department of General Internal Medicine 3, Kawasaki Medical School General Medical Center, Okayama, Japan

**Keywords:** Ventricular Fibrillation, Inflammation, Tachycardia, Ventricular, Myocarditis, Ventricular Premature Complexes

## Abstract

**Background:**

Ventricular parasystole is strongly associated with ventricular fibrillation (VF) in patients with non-ischaemic cardiomyopathy. However, the relationship between ventricular parasystole and cardiac sarcoidosis (CS) remains unclear. The purpose of this study was to examine the prevalence of parasystole in patients with CS.

**Methods:**

This was a retrospective observational study of 214 consecutive patients diagnosed with CS (mean age: 69±12 years, 104 males, median follow-up period: 6.8 years (IQR: 3.2–10.7) in our centre. We investigated parasystole in the patients who developed ventricular arrhythmia (VA) using 9886 ECGs, 280 Holter ECGs and 6391 implantable cardioverter defibrillator interrogation records. Classic parasystole was defined as three ventricular ectopic beats with the same morphology, occurring at integer-multiple intervals but with different coupling intervals (CI) on ECG. New parasystole was defined as two ventricular ectopic beats with a CI difference of more than 120 ms. We also analysed the correlation between inflammation sites and parasystole morphology observed on a 12-lead ECG.

**Results:**

VA was identified in 95 patients (33.7%), and 22 developed VF (23.2%). Parasystole was observed in 12 of the 22 patients with VF (classic: 5, new: 7), 20 of 73 with ventricular tachycardia (classic: 5, new: 15) and 44 of 118 without VA (classic: 16, new: 28). Parasystole was significantly more common in the VF group than in the non-VF group (p=0.049). The site of inflammation observed on ^18^F-fluorodeoxyglucose positron emission tomography performed within 3 months after the development of VA and the origin of parasystole matched in all four patients with VF who had 12-lead ECG records of parasystole. Inflammation was correlated with the origin of parasystole.

**Conclusion:**

Ventricular parasystole was detected in one-third of patients with CS in this study, especially those with VF. The presence of parasystole and inflammation may predict the occurrence of VF in patients with CS.

**WHAT IS ALREADY KNOWN ON THIS TOPIC**

- Ventricular parasystole, characterised by automaticity and entrance block, is a rare finding suggested to be associated with ventricular fibrillation (VF). However, the prevalence of parasystole and its correlation with VF and inflammation in patients with cardiac sarcoidosis (CS) remain unclear.

**WHAT THIS STUDY ADDS**

- Ventricular parasystole in cardiac sarcoidosis is significantly more common in patients with CS who develop VF than in those who do not, and its origin matches the site of inflammation detected on ^18^F-fluorodeoxyglucose positron emission tomography.

**HOW THIS STUDY MIGHT AFFECT RESEARCH, PRACTICE OR POLICY**

- Ventricular parasystole is not rare in patients with cardiac sarcoidosis and is particularly common in those with a history of VF. Given that the origin of ventricular parasystole matches the site of inflammation, evaluating inflammation in patients with CS may be beneficial if parasystole is observed.

## Introduction

 Cardiac sarcoidosis (CS) is a heterogeneous, non-caseating, granulomatous inflammation cardiomyopathy of unknown cause that presents with three main clinical manifestations: conduction system disorder, lethal ventricular arrhythmias (VA) and heart failure.^1^,^3^ Myocardial inflammation could be a critical risk factor for ventricular tachycardia (VT) and ventricular fibrillation (VF) in infiltrative cardiomyopathy.^4 5^

Ventricular parasystole is a rare feature of ventricular ectopic foci wherein a ‘protected’ intrinsic pacemaker allows the coexistence of double or multi-rhythms.^6^ Ventricular parasystole can be described as an electric injury caused by post-myocardial infarction, inflammation, and remodelling.^6^

We hypothesised that in CS, which typically involves localised inflammation, parasystole could be correlated with inflammation, which leads to ventricular arrhythmia. Notably, reports on parasystole in patients with CS are scarce. Therefore, the aim of this study was to investigate the prevalence of ventricular parasystole among patients with CS. In addition, we analysed the clinical relevance of ventricular parasystole in CS.

## Methods

### Study population and diagnostic criteria

This was a retrospective observational study of 214 consecutive patients who were diagnosed with or treated for CS in the National Cerebral and Cardiovascular Center between 1989 and 2021. All the patients met the latest CS guidelines published by the Japanese Circulation Society in 2017.^7^ The patients’ clinical data, including demographic information recorded at the time of the initial CS diagnosis, were collected for the final follow-up.

All the patients consented to receive their treatments, and all the data analysed in this study were anonymised. We obtained informed consent using the opt-out method, which involved posting a leaflet or poster approved by the review committee. This study was conducted in accordance with the principles of the Declaration of Helsinki.

### Definition of parasystole

We reviewed all data from scanned pacemakers, implantable cardioverter defibrillators (ICD) and cardiac resynchronisation therapy (CRT) interrogations. We defined monomorphic VT or VF as all episodes of VT and VF for which the patient either received at least one therapy or episodes that spontaneously terminated after persisting for 30 s or longer. Additionally, we reviewed all data from 12-lead ECGs and Holter monitors available in the patients’ electronic medical records until 31 December 2023.

**Figure 1:**
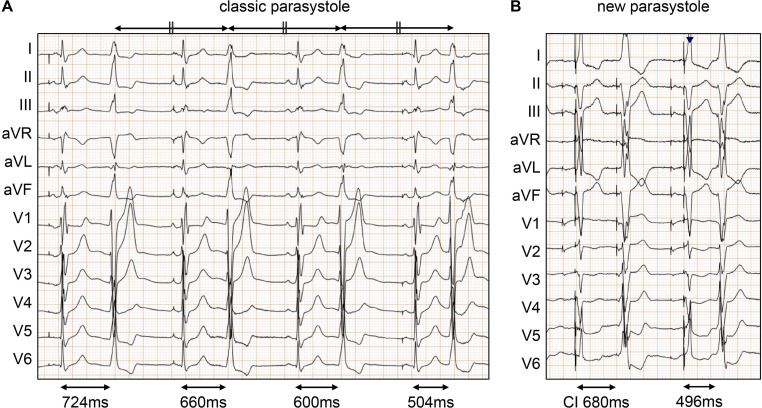
Definition of ventricular parasystole. (**A**) Classic parasystole was defined as PVCs with the same morphology and coupling intervals different from the previous QRS complex within the same continuous ECG recording. At least three PVCs appear at intervals measured using callipers, occurring multiple times in a fixed interval. (**B**) New parasystole was defined as PVCs with the same morphology and higher coupling interval variability than that expected of non-parasystolic PVCs (defined as at least a 120 ms difference between the shortest and longest coupling intervals within the same continuous ECG strip), even if the presence of three beats at a constant interval could not be demonstrated. PVC, premature ventricular contraction.

We defined parasystole according to the definition provided in the study by Do *et al*.^6^ Ventricular parasystole was defined as premature ventricular contractions (PVCs) with the same morphology but coupling intervals different from that of the preceding QRS complex in the same continuous ECG recording. Classic parasystole was defined as PVCs with the same morphology but coupling intervals different from that of the previous QRS complex within the same continuous ECG recording. At least three premature PVCs appear at intervals measured using callipers, occurring multiple times in a fixed interval (figure 1).^6^ Additionally, parasystole can be defined as PVCs exhibiting higher coupling interval variability than non-parasystolic PVCs, with a difference of at least 120 ms between the shortest and longest coupling interval within the same continuous ECG strip, even if a constant interval of three beats cannot be demonstrated (figure 1).^8^

### Definition of ventricular arrhythmias

VA was defined as sustained VT and VF. Sustained VT was defined as monomorphic cardiac arrhythmia of ≥3 consecutive complexes originating in the ventricles at a rate >100 beats per minute (cycle length: <600 ms) for >30 s or requiring termination due to haemodynamic compromise in <30 s.^9^ VF was defined as a rapid, grossly irregular electrical activity with marked variability in the electrocardiographic waveform, with the ventricular rate typically being >300 beats per minute (cycle length: <200 ms), and polymorphic VT.^9^

### Imaging examination

At the time of diagnosis or during follow-up, we assessed inflammation in all patients with CS using ^18^F-fluorodeoxyglucose positron emission tomography (FDG-PET), gallium (Ga) scintigraphy or both. Patients underwent cardiac FDG-PET imaging in a fasting state (>12 hours), with images acquired 1 hour after the injection of 2 MBq/kg body weight of ^18^F-fluoro-2-deoxyglucose.^10 11^ A positive PET scan was defined as a focal, diffuse or focal-on-diffuse pattern of increased tracer uptake in the myocardium. Ga scintigraphy was performed 48 hours after injection of ^67^Ga citrate at a dose of 111 MBq. Positive Ga scintigraphy was defined as an accumulation of the tracer in the heart.^11^ Cardiac FDG-PET and Ga uptake were confirmed based on the consensus of experienced radiologists at the National Cerebral and Cardiovascular Center. Baseline imaging (FDG-PET or/and Ga scintigraphy) was performed at the time of CS diagnosis, and the patients were encouraged to undergo serial FDG-PET scans or/and Ga scintigraphy every 6–12 months. Moreover, additional FDG-PET scans were performed for patients who underwent a change in therapy or clinical status. Selection of the imaging modality for evaluating myocardial inflammation was based on each physician’s decision.

### Study protocol

We analysed the correlation between parasystole and CS in patients with and without VA. The patients were categorised into three groups for analysis: VF group, which included patients that showed at least one documented VF event; VT group, which included patients with VT but no documented VF; and non-VA group, which included patients that showed no documented VA during the follow-up period.

We investigated the association between the origin of parasystolic PVCs and the inflammatory sites observed on FDG-PET/Ga scintigraphy, which were determined by experienced radiologists and two electrophysiologists (TI and KN). We defined positive inflammation as FDG-PET or Ga scintigraphy uptake in the myocardium within 3 months before or after the detection of ventricular parasystoles.

### Statistical analysis

Continuous variables are expressed as mean±SD or median (IQR), whereas categorical variables are presented as numbers and percentages. Pearson χ2 tests were used to compare categorical and continuous variables. All tests were two-tailed, and p<0.05 was considered statistically significant. All analyses were performed using IBM SPSS V.29.0 (IBM SPSS Statistics, IBM Corporation, Armonk, New York).

### Patient and public involvement statement

Neither patients nor members of the public have been or will be involved in the design of the study, its planning or the data collection or data analysis.

## Results

### Characteristics of the patient cohort

We included 214 patients with CS diagnosed based on the Japan Circulation Society 2017 guidelines.^7^ Of the 214 patients, 82 (38.3%) had isolated CS (table 1). For 33 (15.4%) of the patients in the cohort, sarcoidosis was confirmed based on histological findings obtained from endomyocardial biopsy. The clinical characteristics of the patients at the time of CS diagnosis are shown in table 1. The mean age of the patients was 69±12 years and half of them were male. The first clinical cardiac manifestation was VA in 42 (19.6%) patients, VT in 34 (15.9%) and VF in 6 (2.8%). Symptomatic atrioventricular block was the first clinical manifestation in 59 (27.6%) patients. The initial cardiac manifestations in other patients were palpitations, asymptomatic premature ventricular contractions or no symptoms.

**Table 1:** Characteristics of the patient cohort

	Alln=214	VFn=22	VTn=74	Non-VAn=118
Sex: male, n (%)	104 (48.6)	11 (50.0)	40 (54.1)	52 (44.1)
Age (years), mean±SD	69±12	64±13	68±12	70±11
LVDd (mm), mean±SD	57±11	61±12	58±10	56±11
LVDs (mm), mean±SD	45±13	50±14	47±13	43±13
LVEF (%), mean±SD	39±14	36±13	38±14	41±14
JCS criteria for the diagnosis of cardiac sarcoidosis				
Histological diagnosis, n (%)	21 (9.8)	3 (13.6)	5 (6.8)	13 (11.0)
Clinical diagnosis, n (%)	112 (52.3)	8 (27.3)	36 (48.6)	68 (57.6)
Isolated histological diagnosis, n (%)	13 (6.1)	3 (13.6)	3 (4.1)	7 (5.9)
Isolated clinical diagnosis, n (%)	68 (31.8)	8 (36.4)	30 (40.5)	30 (25.4)
Organ involvement				
Lung, n (%)	123 (57.5)	9 (40.9)	37 (50)	77 (65.3)
Eyes, n (%)	21 (9.8)	2 (9.1)	7 (9.5)	12 (10.2)
Skin, n (%)	14 (6.5)	1 (4.5)	5 (6.8)	8 (6.8)
Others, n (%)	15 (7.0)	1 (4.5)	5 (6.8)	8 (6.8)
Advanced imaging				
FDG-PET, n (%)	186 (86.8)	22 (100)	65 (87.8)	99 (83.9)
Ga scintigraphy, n (%)	159 (74.3)	17 (77.3)	54 (73.0)	86 (72.9)
LGE-CMR, n (%)	131 (61.2)	17 (77.3)	42 (56.8)	73 (61.9)
First cardiac manifestations				
Heart failure, n (%)	53 (24.8)	8 (36.4)	18 (24.3)	28 (23.7)
Ventricular arrhythmia, n (%)	42 (19.6)	13 (59.1)	28 (37.8)	0 (0)
Atrioventricular block, n (%)	59 (27.6)	0 (0)	19 (25.7)	40 (33.9)
Others, n (%)	59 (27.6)	1 (4.5)	9 (12.2)	50 (42.4)
Conduction disturbance				
AVB, n (%)	77 (40.0)	7 (31.8)	25 (33.8)	45 (38.1)
CHB	50 (23.4)	1 (4.5)	18 (24.3)	31 (26.3)
Other AVB	27 (12.6)	6 (27.3)	7 (9.5)	14 (11.9)
Intrinsic QRS complex abnormalities, n (%)	93 (43.5)	13 (59.1)	32 (43.2)	48 (40.7)
RBBB only	27 (12.6)	3 (13.6)	8 (10.8)	16 (13.6)
LAFB only	14 (6.5)	4 (18.2)	3 (4.1)	7 (5.9)
LPFB only	3 (1.4)	0 (0)	0 (0)	3 (2.5)
LBBB	8 (3.7)	1 (4.5)	3 (4.1)	4 (3.4)
IVCD	3 (1.4)	1 (4.5)	0 (0)	2 (1.7)
RBBB+LAFB	35 (16.4)	3 (13.6)	17 (23.0)	15 (12.7)
RBBB+LPFB	2 (0.9)	1 (4.5)	0 (0)	1 (0.8)
Trifascicular block	1 (0.5)	0 (0)	1 (1.4)	0 (0)
Treatment				
Steroid administration, n (%)	175 (81.8)	21 (95.5)	66 (89.2)	88 (74.6)
Defibrillator implantation, n (%)	132 (61.7)	22 (100)	65 (87.8)	45 (38.1)
Primary prevention, n (%)	68 (31.8)	4 (18.2)	19 (25.7)	45 (38.1)
Secondary prevention, n (%)	64 (29.9)	18 (81.2)	46 (62.2)	0 (0)
CRT implantation, n (%)	48 (22.4)	2 (9.1)	14 (18.9)	32 (27.1)

Values are mean SD or n (%) unless otherwise indicated.

AVB, atrioventricular block; CHB, complete heart block; CMR, cardiac MRI; CRT, cardiac resynchronization therapy; FDG-PET, 18-fluoro-deoxyglucose positron emission tomography; Ga, garium; IVCD, intraventricular conduction disturbance; JCS, Japan Circulation Society; LAFB, left anterior fascicular block; LBBB, left bundle branch block; LGE-CMR, late-gadolinium enhancement-CMR; LPFB, left posterior fascicular block; LVDd, left ventricular end-diastolic diameter; LVDs, left ventricular end-systolic diameter; LVEF, left ventricular ejection fraction; RBBB, right bundle branch block; VA, ventricular arrhythmia; VF, ventricular fibrillation; VT, ventricular tachycardia.

The median follow-up duration was 6.8 years (IQR: 3.2–10.7). During follow-up, 74 (34.6%) patients had documented VT alone, 9 (4.2%) had VF alone and 13 (6.1%) had both VT and VF. However, initiation of VF or VT was not recorded in the devices and 12-lead ECGs. ICDs or CRT-Defibrillators were implanted in 132 patients (61.7%) as a secondary option for the prevention of VF (81.8%) and VT (62.2%). A total of 175 patients (81.8%) received steroids for active cardiac inflammation.

### Prevalence of parasystole

Data from 9886 12-lead ECGs and 280 Holter monitors were reviewed in this study. The median number of 12-lead ECGs per patient was 37, and 123 (57.7%) patients had Holter ECGs.

Ventricular parasystole was identified in 76 patients (35.7%). Of these, 26 (12.2%) had classic parasystole and 50 (23.5%) had new parasystole, which were determined based on the presence of PVCs with coupling interval variability>120 ms) (table 2, figure 1). New parasystole was observed in all cases of classic parasystole. Separate analyses of parasystole recorded on both Holter ECG and 12-lead ECG revealed that Holter ECG detected significantly more cases of parasystole than 12-lead ECG (25.7% vs 15.4%, p<0.01).

**Table 2:** Prevalence of parasystole

	Alln=214	VFn=22	VT onlyn=74	VAn=96	Non-VAn=118
Classic parasystole, n (%)					
Total	26 (12.1)	5 (22.7)	5 (6.8)	10 (10.4)	16 (13.6)
12 ECG	10 (4.7)	3 (13.6)	3 (4.1)	6 (6.3)	4 (3.4)
Holter ECG	16 (7.5)	2 (9.1)	2 (2.7)	4 (4.2)	12 (10.2)
New parasystole, n (%)					
Total	50 (23.4)	7 (31.8)	15 (20.3)	22 (22.9)	28 (23.7)
12 ECG	23 (10.7)	3 (13.6)	6 (8.1)	9 (9.4)	14 (11.9)
Holter ECG	27 (12.6)	4 (18.2)	9 (12.2)	13 (13.5)	14 (11.9)
Classic+new parasystole, n (%)					
Total	76 (35.5)	12 (54.5)	20 (27.0)	32 (33.3)	44 (37.3)
12 ECG	33 (15.4)	6 (27.3)	9 (12.2)	15 (15.6)	18 (15.3)
Holter ECG	43 (20.0)	6 (27.3)	11 (14.9)	17 (17.7)	26 (22.0)

12 ECG, 12 lead electrocardiogram; Holter ECG, Holter electrocardiogram; VA, ventricular arrhythmia; VF, ventricular fibrillation; VT, ventricular tachycardia.

Although no significant correlation was observed between parasystole and VT, the presence of parasystole was significantly associated with the occurrence of VF (12 (16%) vs 10 (7%), p=0.049, table 3). Notably, parasystole was inversely correlated with VT and was more common in patients without VT than in those with VT (20 (26%) vs 54 (39%), p=0.059, table 3). Atrioventricular block (AVB) was observed in 77 (40.0%) patients, including 50 (23.4%) with complete AVB. There was no significant difference in the occurrence of AVB between patients with and without parasystole. Bifascicular block with right bundle branch block (RBBB) and left anterior fascicular block were the most common findings in patients with abnormal intrinsic QRS complex, followed by RBBB (27 (12.6%)). During follow-up, 22.9% of the patients showed progression of conduction disturbance regardless of whether parasystole was detected or not (table 3).

**Table 3:** Characteristics of patients with and without parasystole

	Parasystole (+)n=76	Parasystole (−)n=138	P value
Age, years	68±11	69±12	0.18
Male, n (%)	30 (39)	74 (54)	0.047
LVDd, mm	58±13	56±10	0.24
LVDs, mm	47±15	45±13	0.31
LVEF, %	37±14	40±14	0.15
JCS criteria for the diagnosis of cardiac sarcoidosis			
Histological diagnosis, n (%)	9 (12)	12 (9)	0.46
Clinical diagnosis, n (%)	40 (53)	73 (53)	0.97
Isolated histological diagnosis, n (%)	7 (9)	6 (4)	0.15
Isolated clinical diagnosis, n (%)	22 (30)	47 (34)	0.44
Ventricular arrhythmia			
VF, n (%)	12 (16)	10 (7)	0.049
VT, n (%)	20 (26)	54 (39)	0.059
Non-VA, n (%)	44 (58)	74 (54)	0.55
Conduction disturbance			
AVB, n	30 (39)	47 (34)	0.43
CHB	18 (24)	32 (23)	0.93
Other AVB	12 (16)	15 (11)	0.30
Intrinsic QRS complex abnormalities, n	38 (50)	62 (45)	0.48
RBBB only	10 (13)	17 (12)	0.86
LAFB only	3 (4)	8 (6)	0.56
LPFB only	0 (0)	2 (1)	0.29
LBBB	2 (3)	5 (4)	0.70
IVCD	2 (3)	1 (1)	0.26
RBBB+LAFB	15 (20)	19 (14)	0.25
RBBB+LPFB	1 (1)	1 (1)	0.67
Trifascicular block	0 (0)	1 (1)	0.46
QRS complex morphology changes, n	22 (29)	27 (20)	0.12
Progressive IVCD	0 (0)	4 (3)	0.13
New RBBB	2 (3)	3 (2)	0.83
New LAFB	3 (4)	3 (2)	0.45
New LPFB	0 (0)	0 (0)	–
New LBBB	2 (3)	0 (0)	0.056
New CHB	8 (11)	12 (9)	0.66
New other AVB	7 (9)	5 (4)	0.09
No change	54 (71)	109 (79)	0.19
Not available	0 (0)	2 (1)	0.29
Treatments			
Steroid administration, n (%)	65 (86)	110 (80)	0.29

Values are mean SD or n (%) unless otherwise indicated.

AVB, atrioventricular block; CHB, complete heart block; IVCD, intraventricular conduction disturbance; JCS, Japan Circulation Society; LAFB, left anterior fascicular block; LBBB, left bundle branch block; LPFB, left posterior fascicular block; LVDd, left ventricular end-diastolic diameter; LVDs, left ventricular end-systolic diameter; LVEF, left ventricular ejection fraction; RBBB, right bundle branch block; VA, ventricular arrhythmia; VF, ventricular fibrillation; VT, ventricular tachycardia.

### Ventricular parasystole and ventricular arrhythmias

63 patients had parasystole. Of these, 12 (5.6%) developed VF and 20 (9.4%) developed VT. 5 of the 12 (41.6%) patients who developed VF had classic parasystole, and 5 of the 20 (25%) who developed VT had classic parasystole (table 3). The estimated origins of parasystole based on 12-lead ECG findings in the VF, VT and non-VA groups are summarised in table 4. 35 parasystole waveforms were recorded on the 12-lead ECGs, and their locations were estimated. Two patients exhibited two different types of parasystole. Many waveforms appeared to be septal in origin, with or without VA (VA vs non-VA=9 (57%) vs 13 (68%)). However, parasystole that resulted in VA was more common in the left anterior fascicular branch region than that which did not result in VA.

**Table 4:** Parasystole PVC details

	VF group	VT only	VA	Non-VA
Parasystolic foci (n=35)				
1	6	8	14	17
2	0	1	1	1
PVC origin (n=35)	6	10	16	19
LV anterior	0	0	0	1 (5%)
LV anteroseptum	1 (17%)	3 (30%)	4 (25%)	6 (32%)
LV posteroseptum	1 (17%)	0	1 (6%)	4 (21%)
LV posterior	0	0	0	0
LV posterolateral	1 (17%)	0	1 (6%)	0
LV anterolateral	2 (33%)	4 (40%)	6 (38%)	3 (16%)
RV septum	1 (17%)	3 (30%)	4 (25%)	5 (26%)

LV, left ventricle; PVC, premature ventricular contraction; RV, right ventricle; VA, ventricular arrhythmia; VF, ventricular fibrillation; VT, ventricular tachycardia.

**Figure 2:**
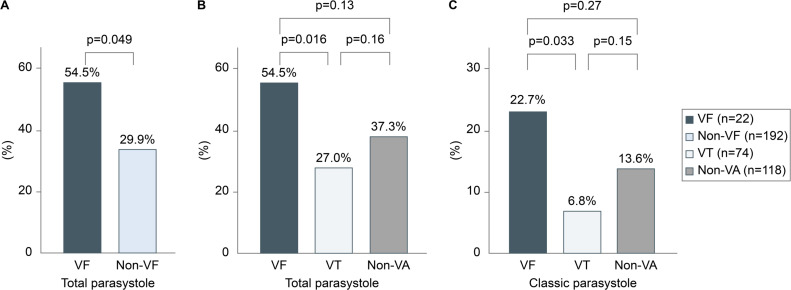
Prevalence of parasystole in each group. (**A**) Total prevalence (classic and new) of parasystole in the VF and non-VF groups. (**B**) Total prevalence of parasystole in the VF, VT and non-VA groups. (**C**) Prevalence of classic parasystole in the VF, VT and non-VA groups. VA, ventricular arrhythmia; VF, ventricular fibrillation; VT, ventricular tachycardia.

Parasystole was significantly more common in the VF group than in the non-VF group (54.5% vs 29.9%; p=0.049). 12 of the 22 patients who developed VF and 20 of the 74 who developed VT showed parasystole (54.5% vs 27.0%; p=0.016) (figure 2B). When the analysis was limited to classic parasystole, parasystole was observed in five of the patients in the VF group and five in the VT group (22.7% vs 6.8%; p=0.031) (figure 2C). However, there was no significant difference in the occurrence of parasystole between either group and the non-VA group (total: 54.5% vs 37.3%, p=0.12; classic: 22.7% vs 13.6%, p=0.27; figure 2B,C). Analysis conducted using data on classic parasystole observed in 12-lead ECGs revealed that the incidence of parasystole in the VF group was significantly higher than that in the VT group (13.6% vs 4.1%; p=0.006) and the non-VA group (13.6% vs 3.4%; p=0.043). In the VF group, parasystole was recorded within 3 months after the occurrence of VA in 5 out of 12 cases, either on a 12-lead ECG or Holter ECG. In the VT group, parasystole was documented within 3 months after the occurrence of VT in 5 out of 20 cases. Furthermore, ventricular parasystole was detected within a median time of 49 days (IQR: −284 to 872) of occurring ventricular arrhythmias, 10 of which occurred before and 22 days after VA. In particular, 17 patients (53%) had parasystole within a year before and after the detection of ventricular arrhythmias.

### Ventricular parasystole and inflammation

**Figure 3:**
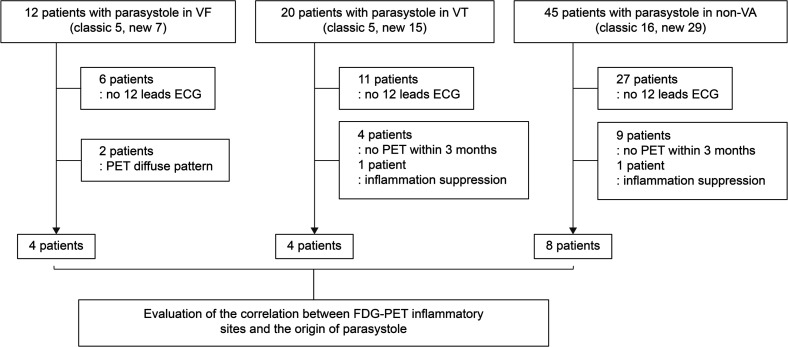
Flowchart of the identification of the patients who had data on the identification of inflammatory sites and origin of parasystole on FDG-PET. Parasystole was observed in 12 patients in the VF group (classic: 5, new: 7), 20 patients in the VT group (classic: 5, new: 15) and 45 patients in the non-VA group (classic: 16, new: 29). Among these, patients without 12-lead ECG data were excluded (6 in the VF group, 11 in the VT group and 27 in the non-VA group). Additionally, two patients in the VF group who showed diffuse PET uptake were excluded. Four and nine patients in the VT and non-VA groups, respectively, did not undergo PET scans within 3 months. In addition, one patient from each group showed no inflammation on FDG-PET. Finally, the relationship between the site of inflammation detected on FDG-PET and the origin of parasystole was evaluated in four patients in the VF group, four in the VT group and eight in the non-VA group. FDG-PET, ^18^F-fluorodeoxyglucose-positron emission tomography; VA, ventricular arrhythmia; VF, ventricular fibrillation; VT, ventricular tachycardia.

**Figure 4:**
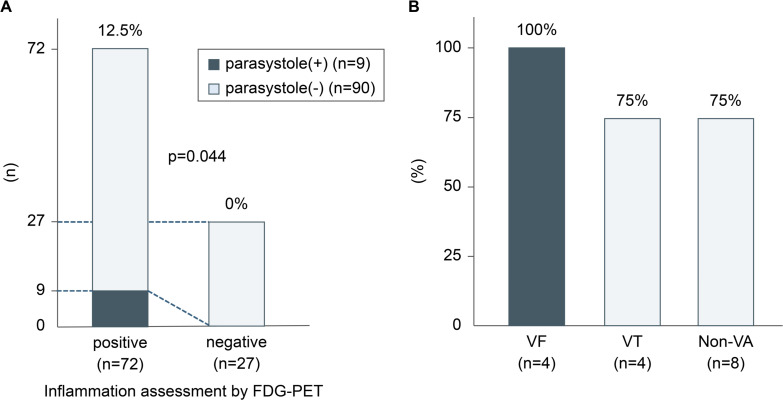
(**A**) Correlation between the timing of parasystole and the period of inflammation detected on FDG-PET in patients with VF. (**B**) Origin of premature ventricular contractions of a parasystole matching the inflammatory site detected on FDG-PET. FDG-PET, ^18^F-fluorodeoxyglucose-positron emission tomography; VA, ventricular arrhythmia; VF, ventricular fibrillation; VT, ventricular tachycardia.

For patients in the VF group in whom the origin of parasystole was identified using a 12-lead ECG and FDG-PET indicated inflammation within 3 months, the site of the FDG-PET-detected inflammation matched the origin of parasystole in all cases (4/4). In contrast, this consistency was observed in most, but not all, cases in the VT group (3/4) and the non-VA group (6/8) (figures3  4B). A significant correlation between parasystole detected on 12-lead ECG and Holter ECG and myocardial FDG uptake not limited to specific sites was observed in 9 out of 72 patients (p=0.044) (figure 4A). VT morphology induced by the catheter matched the recorded parasystole waveform in only one patient in the VT group (online supplemental figure). However, a direct relationship between the parasystole and initiation of VF could not be established.

## Discussion

In this study, we evaluated the prevalence of ventricular parasystole in patients with CS. To our knowledge, this study is the first to demonstrate a correlation between parasystole and VF in patients with CS. Additionally, the findings of this study suggest a potential association between parasystole in patients with a history of VF and sites of inflammation in CS. Furthermore, parasystole was more frequently observed in the VF group than in the non-VF group. Moreover, all the patients showed FDG uptake that corresponded to the timing of the parasystole. Notably, the origin of the parasystole estimated from 12-lead ECGs corresponded to the PET-detected inflammation site in every patient who had 12-lead ECG data on the detection of parasystole morphology.

### Prevalence of parasystole in cardiac sarcoidosis

Parasystole is a rare finding, with a prevalence of approximately 0.1% in the general population.^6 12^ Classic parasystole and ventricular parasystole, including new parasystole, detected on 12-lead ECG or Holter ECG are present in 6% and 9% of patients with structural heart disease, respectively, including 70% of those with non-ischaemic heart disease.^6^ In the present study, which involved the analysis of patients with CS alone, classical parasystole was observed in 13% of the cohort, whereas ventricular parasystole was detected in approximately 40%, suggesting a higher detection rate. This finding may be attributed to the fact that the patients underwent repeated 12-lead ECGs or Holter ECGs. Routine Holter ECGs are rarely performed for asymptomatic patients. However, they are often used to evaluate patients, even asymptomatic or palpitating patients, who show frequent PVCs on 12-lead ECGs, likely leading to a higher detection rate.

In cases of classic parasystole, at least two out of three or more premature contractions have coupling intervals of more than 120 ms, meaning that classic parasystole also meets the definition of new parasystole. Although the detection rates for both classic and new parasystole in the VF group were not significantly different (23% vs 32%, p=0.50), new parasystole was detected significantly more frequently in the non-VF group (11% vs 22%, p<0.01). This suggests that applying the definition of new parasystole in cases of VF may be meaningful for improving detection rates. Another reason for the higher detection rate may be that CS is a disease-causing conduction disorder. A previous study^6^ indicated that the occurrence of parasystole is dependent on a certain degree of progression of His-Purkinje conduction disturbance, which was detected in approximately 10% of the study’s cohort. In contrast, more than 30% of the patients in the present study already had conduction disturbances, most with complete AVB. As a high proportion of the patients in the present study had complete AVB, it was not possible to assess the progression of the distal side of the His-Purkinje conduction system, which may explain why no difference in the progression of conduction impairment was observed between patients with and without parasystole.

### Relationship between parasystole and inflammation

Parasystole requires an entrance block at the parasystolic focus and automaticity at the exact location.^13^ Although no established mechanism underlying the association between parasystole and inflammation during the inflammatory phase of CS has been identified, it should be noted that VA may present as PVCs, PVC-triggered VF or focal VT due to triggered activity or increased automaticity.^14^ Additionally, steroid therapy that suppresses inflammation reduces PVCs and non-sustained VT in the early stage of inflammation in CS.^15^ This suggests that increased automaticity in the inflamed regions observed on PET may have contributed to the high prevalence of parasystole observed in the present study. However, the VF group had more inflammation at baseline, despite undergoing treatment with more steroids, as in the present study. This may reflect an intervention against intense inflammation, or as described in the previous reports,^4^ it may be that steroid treatment during the inflammatory phase can induce arrhythmias. Based on these findings, it is suggested that patients with a history of VF who undergo steroid treatment may exhibit PET positivity and a higher detection rate of parasystolic PVCs.

In patients with VF who showed parasystole on a 12-lead ECG within 3 months after an FDG-PET scan, the site of inflammation detected on the PET scan matched the estimated origin of the parasystole (figure 4B). In contrast, although significant concordance was noted in the VT and non-VA groups, it was inconsistent across patients (figure 4B). These findings are consistent with previous reports that suggest a link between VF and parasystole.^6^ In this study, two primary mechanisms may account for the observed association between VF and parasystole, while no such correlation was found with VT. (1) A parasystolic PVC with a short coupling interval leads to an R-on-T phenomenon and triggers VF. (2) With a parasystolic PVC, atrial pacing within the implanted device’s blanking period and the subsequent ventricular pacing may coincide with an intrinsic R wave or the R wave of the PVC, leading to an R-on-T phenomenon and increasing the risk of VF.^6^ Analysis of Holter ECG data indicated that all the patients with VF showed elevated FDG uptake in the myocardium during the periods when parasystole was recorded. Due to the frequent ECG monitoring after hospitalisation for VA, parasystole was naturally more likely to be detected after VA. However, these findings suggest that the presence of parasystole could indicate the need for reassessment of inflammation in patients with a history of VF, especially after 1 year.

### Limitations

This was a single-centre retrospective observational study. Although the a priori prevalence of ventricular parasystole is low, it should be noted that it may be missed in patients with outpatients and in patients with fewer hospitalisations and ECGs. In the VF group, fewer patients had more than four Holter ECG recordings compared with the non-VA group. Among the VF group, three patients had more than four Holter recordings, with parasystole detected in one (33.3%). In contrast, in the non-VA group, 15 patients underwent at least four recordings, and parasystole was detected in 14 cases (93.3%) (VF vs non-VA=33.3% vs 93.3%; p=0.01). Since parasystole can vary during the day, capturing it using only a 12-lead ECG is difficult. Additionally, although parasystole was frequently recorded during periods when VF was observed, parasystole was not consistently documented around VF events. It is possible that parasystole was not recorded due to under-recognition or because VAs did not completely correspond to the timing of parasystole. Previous evidence^16^ indicates that FDG uptake is no longer visible in PET scans acquired 1 month after initiating steroid treatment, suggesting that steroid therapy may weaken the association between parasystole and inflammation.

## Conclusions

In this study, ventricular parasystole was present in more than one-third of patients with CS, suggesting parasystole is not rare in patients with CS. In addition, the results of this study indicate that parasystole is more common in patients with CS and VF than in those without VF. In patients with VF, the origin of ventricular parasystole observed on recorded 12-lead ECG matches the sites of inflammation observed in the most recent PET. These findings indicate that parasystole is associated with VF and may be a marker of inflammation in CS.

## Supplementary material

10.1136/openhrt-2025-003196 online supplemental file 1

10.1136/openhrt-2025-003196 online supplemental file 2

## Data Availability

All data relevant to the study are included in the article or uploaded as supplementary information.
