# Supplementary figures and images for: Prevalence of ventricular parasystole in patients with cardiac sarcoidosis: correlation between parasystole and inflammation in ventricular fibrillation
Source: Open Heart. 2025 Apr 9;12(1):e003196. doi: 10.1136/openhrt-2025-003196 (PMC11987139; doi:10.1136/openhrt-2025-003196)

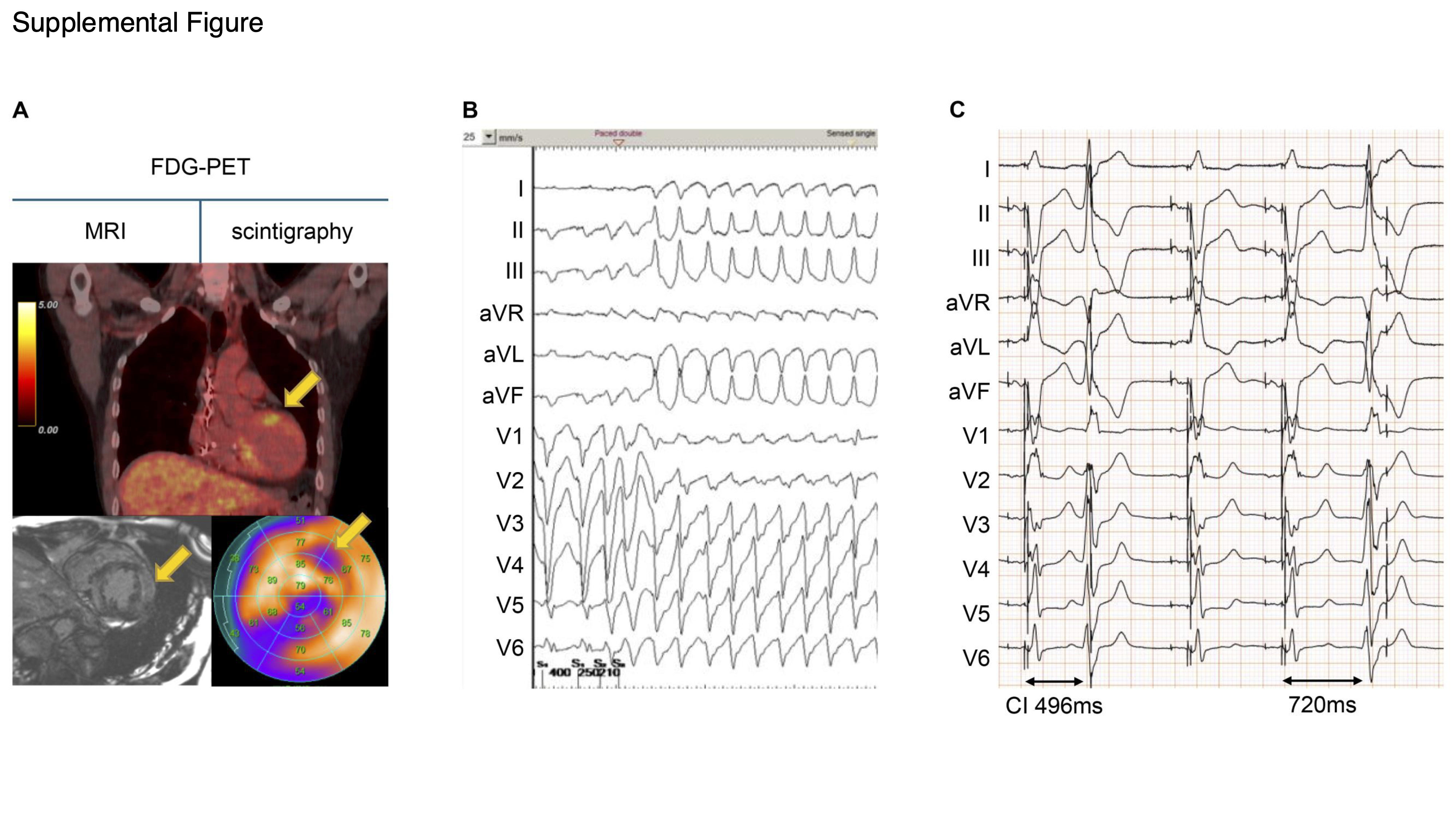

Supplement: online supplemental file 1 [file openhrt-12-1-s001.tiff]
