# Supplementary material for: Prevalence of ventricular parasystole in patients with cardiac sarcoidosis: correlation between parasystole and inflammation in ventricular fibrillation
Source: Open Heart. 2025 Apr 9;12(1):e003196. doi: 10.1136/openhrt-2025-003196 (PMC11987139; doi:10.1136/openhrt-2025-003196)
Supplement: online supplemental file 2 [file openhrt-12-1-s002.docx]

**Supplemental Figure. VT with the same morphology as ventricular parasystole**

A man in his 60s with complete atrioventricular block received a pacemaker. Over time, he showed a decline in cardiac function (LVEF decreased from 55% to 30%), thinning of the basal septum, and wall motion abnormalities from the septum to the inferior wall. MRI showed LGE in the same region as FDG-PET uptake, specifically in the inferior septum and basal-mid anterolateral wall. In addition, perfusion defects in the same areas were identified on scintigraphy (Supplemental Figure A, yellow arrowhead). A myocardial biopsy confirmed cardiac sarcoidosis. During an EPS study, VT was induced with a double extra stimulus (400/250/210 ms) from the RV apex (Supplemental Figure B), consistent with the morphology of the parasystole morphology (Supplemental Figure C).

EPS, electrophysiology study; FDG-PET, ^18^F-fluorodeoxyglucose-positron emission tomography; LGE-CMR, late-gadolinium enhancement-cardiac magnetic resonance imaging; LVEF, left ventricular ejection fraction; RV, right ventricle; VT, ventricular tachycardia
